# An association between time-varying serum alkaline phosphatase concentrations and mortality rate in patients undergoing peritoneal dialysis: a five-year cohort study

**DOI:** 10.1038/srep43314

**Published:** 2017-03-03

**Authors:** Ying Liu, Jin-Gang Zhu, Ben-Chung Cheng, Shang-Chih Liao, Chih-Hsiung Lee, Wen Xiu Chang, Jin-Bor Chen

**Affiliations:** 1 Department of Nephrology, Tianjin First Center Hospital, Tianjin, CO300192, China; 2 Department of Nephrology, Tianjin Union Medicine Center, Tianjin, CO300000, China; 3 Division of Nephrology, Department of Internal Medicine, Kaohsiung Chang Gung Memorial Hospital, Chang Gung University College of Medicine, CO833, Taiwan; 4 Division of Nephrology, Kaohsiung Municipal Feng-Shan Hospital, CO830, Taiwan

## Abstract

The relationship between serum alkaline phosphatase (ALP) concentrations and mortality in peritoneal dialysis (PD) patients is rarely reported. We enrolled 667 PD patients in one PD centre in Taiwan to retrospectively examine the association between three ALP concentrations (baseline, time-averaged, time-dependent) and mortality over a 5-year period (2011–2015). Baseline data collection included demographics, clinical, and laboratory parameters. Multivariable-adjusted Cox models were used to analyse the association. Four ALP quartiles were defined at the baseline: ≤62, 63–82, 83–118, and ≥119 U/L. Of 667 patients, 65 patients died, of which 8 patients died due to cardiovascular disease. Females were predominant in the higher ALP quartiles, and 24-h urine volume was significantly proportionately decreased in the higher ALP quartiles. ALP quartiles expressed by the three models were not associated with all-cause or cardiovascular mortalities after adjusting for demographics, liver function, bone metabolism, mortality, hemoglobin, and 24-h urine volume. In conclusion, ALP concentrations were not associated with death risk in PD patients over the 5-year period.

Serum alkaline phosphatase (ALP) is generally used as an indicator of hepatic and bone diseases owing to its convenient measurement. In chronic kidney disease (CKD) patients, serum ALP concentration is commonly used as a surrogate marker for renal bone disease and hyperparathyroidism. Generally, an elevated serum ALP concentration indicates high-turnover bone disease in CKD. Recently, mounting evidence demonstrates that higher serum ALP concentrations are associated with higher risks for mortality not only in the general population, but also in CKD, including dialysis patients. A report from the National Health and Nutrition Examination Survey in the general US population revealed a graded, independent association between higher ALP levels and increased mortality^1^. Similarly, reports from pre-dialysis CKD and dialysis studies have shown that higher serum ALP levels are associated with increased all-cause mortality^2^,^3^,^4^,^5^,^6^,^7^,^8^,^9^,^10^.

There are only a few reports of the association between serum ALP concentrations and mortality in peritoneal dialysis (PD) patients. One large cohort study from the US, with a median follow-up of 2.7 years, revealed that higher baseline ALP concentrations are associated with increased mortality among PD patients^10^. Another cohort study in a single Chinese centre revealed that the highest baseline ALP quartile was associated with a hazard ratio for all-cause and cardiovascular mortalities with a median follow-up period of 31-months^9^. Owing to dynamic changes in serum ALP concentrations in dialysis patients, we used three ALP concentrations—baseline, time-averaged (TA), and time-dependent (TD)—to examine the associations with mortality in PD patients over a 5-year period.

## Results

Baseline demographics showed a female predominance, especially in ALP concentrations higher than 83 U/L quartiles. Daily urine amounts demonstrated a proportionate decrease as ALP concentrations increased. Dialysis vintage was significantly longer in the highest ALP quartile (Table 1). Laboratory parameters showed significant differences between ALP quartiles and circulating leukocyte count (WBC), glucose, blood urea nitrogen (BUN), creatinine (Cr), albumin-corrected calcium (Ca), sodium (Na), intact parathyroid hormone (iPTH) levels, and cardio-thoracic (CT) ratio (Table 2). The clinical characteristics in the study cohort were expressed in the Supplementary Table 1 and the Supplementary Table 2.

A total of 65 all-cause deaths and 8 cardiovascular (CV) deaths were observed in the study period. Other death causes were infectious diseases (30), malignancy (3), cerebral stroke (3), unknown reason and multi-organ involvement (21). Death risk was not increased for ALP quartiles expressed by baseline, TA, or TD levels in unadjusted analysis (Tables 3 and 4). Similarly, ALP quartiles were not associated with death risk by adjusted model (Tables 5 and 6). Age expressed by baseline, TA, and TD values demonstrated significantly increased all-cause mortality after adjusting for sex, age, 24-h urine volume, hemoglobin, albumin, aspartate aminotransferase (AST), and alanine transaminase (ALT) (model 2). This trend still existed with further adjustment for mineral values (serum Ca, P, iPTH) (Tables 5 and 7). Increases in serum albumin level by 1 gm/dL (baseline, TA, TD values) showed reduced death risk for all-causes (hazard ratio [HR] 0.19, 95% confidence interval (CI) 0.10–0.36, p < 0.001 at baseline; HR 0.12, 95% CI 0.06–0.26, p < 0.001 at TA; HR 0.19, 95% CI 0.10–0.36, p < 0.001 at TD values), and CV mortalities (HR 0.03, 95% CI 0.00–0.31, p = 0.0029 at baseline; HR 0.00, 95% CI 0.00–0.07, p = 0.0002 at TA; HR 0.01, 95% CI 0.00–0.12, p = 0.0004 at TD values) by fully-adjusted analysis (Tables 7 and 8). Reduced serum ALT level by 1 IU/L (baseline and TA values) were also significantly associated with decreased all-cause mortality by fully adjusted analysis (Table 7). Similar results were also demonstrated in the association between time-varying ALT levels (baseline, TA, TD values) and CV mortality (Table 8). Increased albumin-corrected serum Ca levels (TD values) were associated with risk for all-cause mortality by fully adjusted analysis (Table 7). This association disappeared for CV mortality (Table 8). Time-varying iPTH levels were not significantly associated with all-cause and CV mortalities (Tables 7 and 8).

## Discussion

We examined the association between time-varying ALP concentrations for five years and death risk in one of the largest PD centres in Taiwan. Results in the present study did not demonstrate an association between time-varying ALP concentrations and all-cause and CV mortalities. Our result is contrary to the reports from two retrospective studies in PD patients^9^,^10^. Liu *et al*. examined the association between baseline ALP concentration and mortality in incident PD patients for a median 31-month follow-up^9^. They found that the highest ALP quartile was significantly associated with a hazard ratio for all-cause mortality and CV mortality after adjusting for demographics, comorbid conditions, liver function, and bone metabolism parameters. Similarly, Rhee *et al*. examined the association between time-averaged ALP concentration and mortality for at least 3-years of follow-up in a large cohort of PD patients^10^. The results revealed ALP concentrations exceeding 150 U/L were associated with increased mortality (reference ALP: 70 to < 90 U/L). In the present study, we used three models (baseline, time-averaged, time-dependent) to examine the association of ALP concentrations with mortality in PD patients over a 5-year period. We speculate that the different models of ALP concentrations may contribute to the variable results of ALP association with mortality in PD patients. Of note, a further study is needed to clarify this association in PD patients.

In the present study, we found women were predominant in various strata of ALP concentrations. The sex distribution is similar to prior reports^6^,^7^, where female subjects were reported with higher serum ALP levels than male subjects. We also found 24-h urinary volume was significantly proportionately decreased in higher ALP quartiles. The exact explanation is not clear. Increased ALP has been demonstrated in vessels obtained from calcified arterioles in CKD^11^. Moreover, ALP is suggested to be linked to vascular calcification through its role in mediating with pyrophosphate^12^,^13^. Finally, the ALP-death relationship may possibly be related to inflammation and osteomalacia. The latter may result from 25-hydroxyvitamin D deficiency, an independent risk factor for inflammation and CV disease^14^. Peripheral WBC count was found to be significantly increased in higher ALP quartiles in the present study. Therefore, we speculate ALP-associated angiopathy and inflammation may impact upon residual renal function and result in reduced urinary volume. Nevertheless, the relationship between ALP concentrations and residual renal function in PD patients warrants further study.

Our results also demonstrated iPTH concentrations were significantly proportionately increased in higher ALP quartiles. In general, ALP is considered an indicator of high turn-over bone disease in CKD patients^15^. It is reasonable to see the parallel increase in ALP and iPTH concentrations in our cohort. iPTH concentrations in the present study show a neutral effect on mortality in PD patients after fully adjusted analysis. Similarly, other CKD-MBD marker (Ca, P) concentrations were not demonstrated to be associated with death risk in PD patients in the present study. In prior studies, CKD-MBD markers (Ca, P, iPTH) have been reported to be linked to death risk in CKD patients^10^,^16^,^17^,^18^,^19^,^20^,^21^. In the present study, the primary purpose was to examine the relationship between ALP concentrations and mortality in PD patients. The present study was not designed to examine the association between CKD-MBD markers and death risk in PD patients. Therefore, our study could not refute prior studies reporting a relationship between CKD-MBD markers and death risk in CKD patients. Of note, the associations between CKD-MBD markers and ALP concentrations with death risk in PD patients needs further examination in future studies.

Our study has some limitations. First, it was designed as a retrospective study, and all selected subjects were treated at one PD centre; thus, centre-specific effects cannot be excluded. Second, the diversity in clinical practice for different nephrologists could result in non-homogenous management in PD patients and that may result in different outcomes for death. Third, we did not measure bone-specific ALP concentrations. Few studies showed bone-specific ALP was not able to optimally distinguish the ALP isoenzyme^22^,^23^. One study reported that high concentrations of bone-specific-ALP were strongly associated with short-term (6-month) mortality in dialysis patients^24^. However, another study from NHANES data revealed no association between bone-specific ALP concentration and mortality in non-dialysis CKD patients^25^. Finally, the sample size in the present study is relatively small regarding CV mortality. Thus, an underestimation of the association between ALP concentration and CV mortality cannot be avoided.

In conclusion, ALP concentration expressed in baseline, time-dependent, and time-averaged levels, is not associated with mortality in PD patients over a 5-year period. Daily urine amount is proportionately decreased in the higher ALP concentrations in our PD patients.

## Methods

### Subjects

We tracked patients who received PD at Kaohsiung Chang Gung Memorial Hospital in Taiwan from January 1, 2011 to December 31, 2015. By reviewing records, we included patients who were aged ≥18 years at the start of PD. Among them, we excluded patients for the following reasons: discontinuation of PD therapy within 90 days, shifting to haemodialysis, kidney transplantation, or transfer to other hospitals. Finally, a total of 667 prevalent PD patients with a median follow-up 2.72 years were included in the data analysis. All patients underwent PD with varying concentrations of glucose-based PD solutions (1.36%, 2.27%, and 3.86%; Baxter Healthcare SA, Singapore) depending on the prescription of their respective nephrologists. The data review protocol for this study was approved by the Committee on Human Research at the Kaohsiung Chang Gung Memorial Hospital at Taiwan (104–6357B), and the study was conducted in accordance with the principles of the Declaration of Helsinki. Informed consent was not obtained from the patients enrolled in our study, according to the regulation on retrospective data review by the Committee on Human Research at Kaohsiung Chang Gung Memorial Hospital at Taiwan.

### Laboratory measures

Baseline data included age, gender, etiology of kidney failure, history of parathyroidectomy, use of antihypertensives, hepatitis B or C, and 24-h urine volume. Blood examinations at baseline included hemoglobin (Hb), circulating WBC, serum glucose, albumin, aspartate aminotransferase (AST), alanine transaminase (ALT), total bilirubin, ALP, BUN, Cr, Ca, P, Na, potassium, cholesterol, triglyceride, uric acid, ferritin, iPTH. ALP levels were measured in monthly intervals in the study period and recorded for statistical analysis. Corrected serum calcium was calculated by using the following equation: measured total Ca (mg/dL) + 0.8 [4.0 – serum albumin (g/dL)]. All blood parameters were measured using commercial kits and an autoanalyser (Hitachi 7600-210, Hitachi Ltd., Tokyo, Japan). ALP was measured colourimetrically as the hydrolysis of *p*-nitrophenyl phosphate according to instructions from the supplier (Roche Diagnostic Indianapolis, USA). Albumin was measured by the bromocresol green (BCG) method. For the measurement of cardiac-thoracic (CT) ratio, chest radiography was performed after emptying the abdominal cavity of dialysate. Cardiac size was measured by drawing parallel lines on both sides of the heart, at the most lateral points on each side, and measuring the distance between them. Thoracic width was measured by drawing parallel lines down the inner aspect of the widest points of the rib cage, and measuring the distance between these two points. CT ratio is defined as cardiac size/thoracic width.

Standard peritoneal equilibration tests (PETs) were performed at the first, sixth, and twelfth months after PD commencement. Normalised protein catabolic rate (nPCR) was calculated according to K/DOQI clinical practice guidelines for nutrition in chronic renal failure.

### Statistical Methods

#### Demographics

We represent background variables of the study population with number and percentage for categorical data, and means with standard deviations (SD) for continuous data. Demographics included age, gender, and aetiology of kidney failure, antihypertensive agent use, and parathyroidectomy history. We presented the survival events for endpoints prior to the end of the study. Study endpoints were all-cause mortality and CV disease mortality.

### Mortality (Cox model)

We calculated survival time from the time of enrollment to censor or endpoint. Censoring occurred if a patient withdrew from the study, had no event at the end of the study, or was lost to follow-up. Cox proportional hazard models were used to calculate hazard ratios (HRs) for mortality associated with ALP levels only (Model 1). Separate models were created for all-cause and CV mortality. For the unadjusted model 1, baseline ALP levels were treated as a fixed effect, and stratified into four groups: ≤62, 63 to 82, 83 to 118, and ≥119 U/L. Under this assumption, three Cox models were constructed to fit serum ALP levels as baseline level (TB model), or time-averaged serum ALP levels (TA model), or time-dependent serum ALP levels for cohorts (TD model) as the predictor variable, separately. The ALP levels collected in each year were averaged in the TA model; the annual data of ALP per patient was treated as the time-dependent variable in the TD model.

In general, possible covariates adjusted for background characteristics were considered for Model 2, 3. Covariates in model 2 include sex, age, 24-h urinary volume, Hb, albumin, AST and ALT. The final full model (Model 3) adjusted sex, age, 24-h urinary volume, Hb, albumin, AST and ALT, albumin-corrected Ca, P and iPTH.

## Additional Information

**How to cite this article:** Liu, Y. *et al*. An association between time-varying serum alkaline phosphatase concentrations and mortality rate in patients undergoing peritoneal dialysis: a five-year cohort study. *Sci. Rep.* **7**, 43314; doi: 10.1038/srep43314 (2017).

**Publisher's note:** Springer Nature remains neutral with regard to jurisdictional claims in published maps and institutional affiliations.

## Supplementary Material

Supplementary Information

## Figures and Tables

**Table 1:** Summary of demographics and baseline clinical feature.

Characteristic	Alkaline Phosphatase (U/L)								
	≤62		63 to 82		83 to 118		≥119		p-value
	n	%	n	%	n	%	n	%	
Total	175	26.2	165	24.7	161	24.1	166	24.9	
All-Cause Death	11		11		19		24		
CV Death	1		1		4		2		
Age (year)									0.9188
mean, SD	52.4	11.9	51.7	14.5	51.9	14.0	52.7	15.2	
Dialysis Duration (year)									0.0080
mean, SD	2.1	2.5	2.9	4.0	2.7	3.2	3.3	3.5	
Sex									0.0006
Male	71	40.6	87	52.7	76	47.2	52	31.3	
Female	104	59.4	78	47.3	85	52.8	114	68.7	
Etiology of renal failure									0.2270
Glomerulonephritis	96	54.9	88	53.3	92	57.1	86	51.8	
Diabetes mellitus	33	18.9	45	27.3	38	23.6	49	29.5	
Others	46	26.3	32	19.4	31	19.3	31	18.7	
Antihypertensive use									0.8307
Yes	119	68.0	105	63.6	108	67.1	112	67.5	
Parathyroidectomy									0.0532
Yes	11	6.3	16	9.7	12	7.5	24	14.5	
Hepatitis B									0.1195
Yes	32	18.3	19	11.5	16	10.0	25	15.1	
Hepatitis C									0.9710
Yes	12	6.9	13	7.9	11	6.9	13	7.8	
24 hr urinary volume (ml)									<0.0001
mean, SD	0.78	0.72	0.62	0.64	0.56	0.59	0.46	0.57	

Abbreviations: CV, cardiovascular.

**Table 2:** Baseline laboratory data.

Characteristic	Alkaline Phosphatase (U/L)								
	≤62		63 to 82		83 to 118		≥119		p-value
	mean	SD	mean	SD	mean	SD	mean	SD	
Hemoglobin (g/dl)	10.3	1.4	10.3	1.4	10.4	1.5	10.4	1.3	0.6274
WBC (x1000/ul)	6.7	2.3	7.2	2.3	7.2	2.5	7.4	3.0	0.0339
Glucose (mg/dl)	109.2	35.5	122.6	52.3	118.6	44.0	128.1	69.5	0.0072
Albumin (gm/dl)	3.8	0.36	3.81	0.38	3.76	0.40	3.70	0.40	0.0552
AST (IU/L)	21.1	10.7	22.0	13.4	21.6	11.3	23.1	11.8	0.4920
ALT (IU/L)	19.9	13.9	21.0	18.6	21.2	13.8	23.7	16.6	0.1565
T-BIL (mg/dl)	0.38	0.14	0.37	0.16	0.38	0.14	0.35	0.15	0.1246
BUN (mg/dl)	118.8	43.1	115.7	43.4	137.4	91.7	122.7	51.1	0.0064
Creatinine (mg/dl)	11.2	3.1	11.2	3.1	11.1	3.2	10.4	2.7	0.0325
Ca (mg/dl)	9.5	0.7	9.4	0.7	9.4	0.7	9.6	0.8	0.0218
Phosphate (mg/dl)	5.4	1.3	5.3	1.2	5.2	1.3	5.0	1.4	0.0647
Sodium (meq/l)	136.2	4.1	135.5	3.7	135.1	4.5	134.2	4.9	0.0002
Potassium (meq/l)	4.2	0.7	4.1	0.7	4.1	0.7	4.0	0.7	0.2004
Cholesterol (mg/dl)	190.9	47.7	189.1	41.0	187.7	40.6	187.4	47.8	0.8792
Triglyceride (mg/dl)	155.7	115.6	166.6	120.4	154.9	109.3	161.8	109.5	0.7608
Uric acid (mg/dl)	6.8	1.3	7.0	1.4	6.9	1.4	6.9	1.3	0.4279
Ferritin (ng/ml)	412.4	577.8	401.9	368.4	501.9	571.7	454.6	567.0	0.3024
iPTH (pg/ml)	226.4	213.8	248.8	225.5	302.9	298.8	460.3	752.1	< .0001
CTR (%)	0.48	0.06	0.47	0.06	0.48	0.06	0.49	0.06	0.0445
nPCR (gm/Kg/day)	1.03	0.23	1.00	0.22	0.99	0.21	1.03	0.29	0.2410
Kt/V (total)	2.09	0.53	2.09	0.50	2.06	0.42	2.06	0.49	0.8752
Ccr (renal)	26.6	27.1	22.8	28.1	20.4	23.5	13.4	16.2	< .0001
Ccr (total)	69.4	25.8	68.3	25.4	66.2	20.5	63.2	16.4	0.0604

Abbreviations: WBC, leukocyte count; AST, aspartate sminotransferase; ALT, alanine aminotransferase; T-BIL, total bilirubin; BUN, blood urea nitrogen; Ca, albumin-corrected calcium; iPTH, intact parathyroid hormone, CTR, cardiothoracic ratio; nPCR, normalized protein catabolic rate; Ccr, creatinine clearance cc/min/1.73 m^2^/week.

**Table 3:** Hazard ratio of all-cause mortality by serum ALP levels (Model 1).

	HR (95% CI)		
	TB	TA	TD
Alkaline Phosphatase (U/L)			
quartile 1 (≤62)	1.00	1.00	1.00
quartile 2 (63 to 82)	1.00 (0.43 to 2.29)	0.76 (0.31 to 1.87)	0.80 (0.32 to 1.97)
quartile 3 (83 to 118)	1.82 (0.87 to 3.82)	1.13 (0.51 to 2.48)	1.24 (0.57 to 2.69)
quartile 4 (≥119)	2.01 (0.98 to 4.10)	1.28 (0.60 to 2.75)	1.77 (0.85 to 3.69)
P for trend	0.0946	0.5595	0.1425

Cox regression model 1: unadjusted.

Abbreviations: HR, hazard ratio; CI, confident interval.

**Table 4:** Hazard ratio of cardiovascular mortality by serum ALP levels (Model 1).

	HR (95% CI)		
	TB	TA	TD
Alkaline Phosphatase (U/L)			
quartile 1 (≤62)	1.00	1.00	1.00
quartile 2 (63 to 82)	1.04 (0.07 to 16.6)	0.76 (0.05 to 12.1)	0.95 (0.06 to 15.25)
quartile 3 (83 to 118)	4.39 (0.49 to 39.2)	1.86 (0.19 to 17.9)	2.55 (0.26 to 24.74)
quartile 4 (≥119)	2.00 (0.18 to 22.1)	1.70 (0.18 to 16.4)	2.60 (0.27 to 25.27)
P for trend	0.5542	0.8466	0.7053

Cox regression model 1: unadjusted.

**Table 5:** Hazard ratio of all-cause mortality by serum ALP level and clinical feature (Model 2).

	HR (95% CI)		
	TB	TA	TD
Alkaline Phosphatase (U/L)			
quartile 1 (≤62)	1.00	1.00	1.00
quartile 2 (63 to 82)	0.98 (0.42 to 2.30)	0.62 (0.25 to 1.56)	0.73 (0.29 to 1.79)
quartile 3 (83 to 118)	1.74 (0.82 to 3.70)	1.14 (0.50 to 2.60)	1.08 (0.49 to 2.37)
quartile 4 (≥119)	1.63 (0.78 to 3.41)	1.04 (0.46 to 2.36)	1.28 (0.59 to 2.75)
P for trend	0.2829	0.4857	0.5649
Sex			
Male	1.00	1.00	1.00
Female	0.91 (0.54 to 1.54)	0.80 (0.47 to 1.36)	0.90 (0.54 to 1.51)
P for trend	0.7210	0.4088	0.6845
Age (year)			
HR (CI)	1.05 (1.02 to 1.07)	1.02 (1.00 to 1.04)	1.03 (1.01 to 1.05)
P for trend	<0.0001	0.0284	0.0112
24 hr urinary volume			
HR (CI)	0.88 (0.57 to 1.38)	1.49 (0.84 to 2.64)	1.22 (0.76 to 1.93)
P for trend	0.5852	0.1732	0.4096
Hemoglobin (g/dl)			
HR (CI)	0.86 (0.71 to 1.04)	1.00 (0.80 to 1.23)	1.06 (0.89 to 1.26)
P for trend	0.1174	0.9708	0.5073
Albumin (gm/dl)			
HR (CI)	0.18 (0.10 to 0.32)	0.09 (0.05 to 0.18)	0.15 (0.08 to 0.27)
P for trend	<0.0001	<0.0001	<0.0001
AST (IU/L)			
HR (CI)	1.02 (1.00 to 1.04)	1.00 (0.99 to 1.01)	1.00 (1.00 to 1.00)
P for trend	0.0289	0.8241	0.7682
ALT (IU/L)			
HR (CI)	0.97 (0.95 to 1.00)	0.98 (0.96 to 1.00)	0.99 (0.98 to 1.01)
P for trend	0.0263	0.0854	0.4081

Cox regression model 2: adjusted for sex, age, 24-hour urinary volume, hemoglobin, albumin, AST and ALT.

**Table 6:** Hazard ratio of cardiovascular mortality by serum ALP level and clinical feature (Model 2).

	HR (95% CI)		
	TB	TA	TD
Alkaline Phosphatase (U/L)			
quartile 1 (≤62)	1.00	1.00	1.00
quartile 2 (63 to 82)	1.28 (0.08 to 20.9)	1.12 (0.07 to 19.3)	1.29 (0.08 to 21.18)
quartile 3 (83 to 118)	3.12 (0.32 to 30.1)	2.74 (0.21 to 35.3)	1.57 (0.15 to 16.56)
quartile 4 (≥119)	1.76 (0.15 to 20.4)	4.92 (0.38 to 63.4)	2.98 (0.30 to 30.08)
P for trend	0.7285	0.5354	0.7547
Sex			
Male	1.00	1.00	1.00
Female	0.57 (0.12 to 2.71)	0.33 (0.06 to 1.72)	0.39 (0.08 to 1.97)
P for trend	0.4807	0.1886	0.2557
Age (year)			
HR (CI)	1.02 (0.97 to 1.07)	0.99 (0.95 to 1.04)	1.00 (0.96 to 1.05)
P for trend	0.5386	0.6988	0.9496
24 hr urinary volume			
HR (CI)	1.07 (0.29 to 4.00)	4.13 (0.62 to 27.7)	1.78 (0.68 to 4.65)
P for trend	0.9204	0.1440	0.2386
Hemoglobin (g/dl)			
HR (CI)	0.87 (0.50 to 1.50)	0.93 (0.50 to 1.73)	1.07 (0.63 to 1.81)
P for trend	0.6082	0.8178	0.7955
Albumin (gm/dl)			
HR (CI)	0.04 (0.01 to 0.29)	0.01 (0.00 to 0.08)	0.02 (0.00 to 0.13)
P for trend	0.0016	0.0001	0.0001
AST (IU/L)			
HR (CI)	1.00 (0.92 to 1.08)	0.98 (0.89 to 1.07)	1.00 (0.97 to 1.02)
P for trend	0.9162	0.5932	0.6992
ALT (IU/L)			
HR (CI)	0.89 (0.80 to 0.98)	0.84 (0.74 to 0.95)	0.86 (0.76 to 0.97)
P for trend	0.0229	0.0051	0.0112

Cox regression model 2: adjusted for sex, age, 24-hour urinary volume, haemoglobin, albumin, AST and ALT.

**Table 7:** Hazard ratio of all-cause mortality by serum ALP level and clinical feature (Model 3).

	HR (95% CI)		
	TB	TA	TD
Alkaline Phosphatase (U/L)			
quartile 1 (≤62)	1.00	1.00	1.00
quartile 2 (63 to 82)	1.04 (0.45 to 2.44)	0.69 (0.28 to 1.71)	0.78 (0.31 to 1.92)
quartile 3 (83 to 118)	1.92 (0.90 to 4.12)	1.24 (0.53 to 2.87)	1.16 (0.52 to 2.57)
quartile 4 (≥119)	1.88 (0.89 to 3.98)	1.30 (0.56 to 3.05)	1.37 (0.62 to 3.03)
P for trend	0.1691	0.4353	0.5563
Sex			
Male	1.00	1.00	1.00
Female	0.91 (0.53 to 1.56)	0.86 (0.51 to 1.47)	0.93 (0.55 to 1.56)
P for trend	0.7202	0.5897	0.7811
Age (year)			
HR (CI)	1.04 (1.02 to 1.07)	1.02 (1.00 to 1.04)	1.02 (1.00 to 1.04)
P for trend	0.0002	0.0997	0.0496
24 hr urinary volume			
HR (CI)	0.97 (0.61 to 1.52)	1.55 (0.87 to 2.75)	1.25 (0.79 to 1.99)
P for trend	0.8807	0.1379	0.3455
Hemoglobin (g/dl)			
HR (CI)	0.88 (0.73 to 1.07)	0.98 (0.78 to 1.23)	1.02 (0.85 to 1.22)
P for trend	0.2002	0.8585	0.8439
Albumin (gm/dl)			
HR (CI)	0.19 (0.10 to 0.36)	0.12 (0.06 to 0.26)	0.19 (0.10 to 0.36)
P for trend	<0.0001	<0.0001	<0.0001
AST (IU/L)			
HR (CI)	1.02 (1.00 to 1.04)	1.00 (0.99 to 1.01)	1.00 (1.00 to 1.00)
P for trend	0.0277	0.7171	0.8197
ALT (IU/L)			
HR (CI)	0.97 (0.95 to 0.99)	0.97 (0.94 to 0.99)	0.99 (0.97 to 1.01)
P for trend	0.0139	0.0114	0.2006
Ca (mg/dl)			
HR (CI)	1.37 (0.98 to 1.91)	1.52 (1.00 to 2.31)	1.46 (1.06 to 2.02)
P for trend	0.0648	0.0512	0.0215
Phosphate (mg/dl)			
HR (CI)	1.07 (0.87 to 1.32)	0.98 (0.75 to 1.29)	0.91 (0.73 to 1.13)
P for trend	0.5296	0.8993	0.3731
iPTH (pg/ml)			
HR (CI)	1.00 (1.00 to 1.00)	1.00 (1.00 to 1.00)	1.00 (1.00 to 1.00)
P for trend	0.0636	0.0011	0.0216

Cox regression model 3: adjusted for sex, age, 24-hour urinary volume, haemoglobin, albumin, AST and ALT, Ca, Phosphate and iPTH.

**Table 8:** Hazard ratio of cardiovascular mortality by serum ALP level and clinical feature (Model 3).

	HR (95% CI)		
	TB	TA	TD
Alkaline Phosphatase (U/L)			
quartile 1 (≤62)	1.00	1.00	1.00
quartile 2 (63 to 82)	1.63 (0.10 to 27.8)	0.90 (0.05 to 16.8)	1.35 (0.08 to 23.23)
quartile 3 (83 to 118)	3.32 (0.34 to 32.3)	2.61 (0.21 to 32.1)	1.33 (0.12 to 15.10)
quartile 4 (≥119)	2.23 (0.18 to 27.8)	6.00 (0.45 to 80.3)	4.31 (0.38 to 49.52)
P for trend	0.7520	0.3781	0.5441
Sex			
Male	1.00	1.00	1.00
Female	0.79 (0.16 to 3.89)	0.41 (0.08 to 2.17)	0.48 (0.10 to 2.37)
P for trend	0.7690	0.2931	0.3692
Age (year)			
HR (CI)	1.02 (0.96 to 1.07)	0.99 (0.94 to 1.03)	1.00 (0.95 to 1.04)
P for trend	0.5461	0.5601	0.8351
24 hr urinary volume			
HR (CI)	0.91 (0.23 to 3.64)	3.84 (0.51 to 29.1)	1.88 (0.58 to 6.10)
P for trend	0.8990	0.1924	0.2944
Hemoglobin (g/dl)			
HR (CI)	0.82 (0.48 to 1.41)	0.91 (0.43 to 1.88)	1.14 (0.65 to 2.02)
P for trend	0.4810	0.7886	0.6471
Albumin (gm/dl)			
HR (CI)	0.03 (0.00 to 0.31)	0.00 (0.00 to 0.07)	0.01 (0.00 to 0.12)
P for trend	0.0029	0.0002	0.0004
AST (IU/L)			
HR (CI)	1.00 (0.92 to 1.08)	0.99 (0.94 to 1.04)	1.00 (0.98 to 1.01)
P for trend	0.9623	0.6519	0.5897
ALT (IU/L)			
HR (CI)	0.86 (0.76 to 0.98)	0.81 (0.70 to 0.93)	0.83 (0.72 to 0.95)
P for trend	0.0191	0.0031	0.0073
Ca (mg/dl)			
HR (CI)	0.66 (0.26 to 1.65)	0.61 (0.24 to 1.54)	0.62 (0.24 to 1.59)
P for trend	0.3717	0.2944	0.3168
Phosphate (mg/dl)			
HR (CI)	0.87 (0.45 to 1.67)	0.98 (0.46 to 2.05)	1.01 (0.51 to 1.98)
P for trend	0.6762	0.9464	0.9808
iPTH (pg/ml)			
HR (CI)	1.00 (1.00 to 1.00)	1.00 (0.99 to 1.00)	1.00 (0.99 to 1.00)
P for trend	0.6175	0.1824	0.2411

Cox regression model 3: adjusted for sex, age, 24-hour urinary volume, haemoglobin, albumin, AST and ALT, Ca, Phosphate and iPTH.
