# Corrigendum: An association between time-varying serum alkaline phosphatase concentrations and mortality rate in patients undergoing peritoneal dialysis: a five-year cohort study

**DOI:** 10.1038/srep46383

**Published:** 2017-04-25

**Authors:** Ying Liu, Jin-Gang Zhu, Ben-Chung Cheng, Shang-Chih Liao, Chih-Hsiung Lee, Wen Xiu Chang, Jin-Bor Chen

Scientific Reports 7: Article number: 4331410.1038/srep43314; published online: 03 03 2017; updated: 04 25 2017

This Article contains an error in the IRB number. In the Methods section under the subheading ‘Subjects’,

“The data review protocol for this study was approved by the Committee on Human Research at the Kaohsiung Chang Gung Memorial Hospital at Taiwan (104–6357B), and the study was conducted in accordance with the principles of the Declaration of Helsinki”.

should read

“The data review protocol for this study was approved by the Committee on Human Research at the Kaohsiung Chang Gung Memorial Hospital at Taiwan (100–2661B), and the study was conducted in accordance with the principles of the Declaration of Helsinki”.

