# Supplementary material for: An association between time-varying serum alkaline phosphatase concentrations and mortality rate in patients undergoing peritoneal dialysis: a five-year cohort study
Source: Sci Rep. 2017 Mar 3;7:43314. doi: 10.1038/srep43314 (PMC5335666; doi:10.1038/srep43314)
Supplement: Supplementary Information [file srep43314-s1.pdf]

An association between time-varying serum alkaline phosphatase concentrations and mortality rate in patients undergoing peritoneal dialysis: a five-year cohort study

Ying Liu[1], Jin-Gang Zhu[2], Ben-Chung Cheng[3], Shang-Chih Liao[4], Chih-Hsiung Lee[3], Wen Xiu Chang[1], Jin-Bor Chen[3*]

Supplementary Table 1 Summary of demographics and baseline clinical feature in study cohort

	n	%
Total	667	
All-Cause Death	65	
CV Death	8	
Age (year)		
mean, SD	52.2	13.9
Dialysis Duration (year)		
mean, SD	2.7	3.4
Sex		
Male	286	42.9
Female	381	57.1
Etiology of renal failure		
Glomerulonephritis	362	54.3
Diabetes mellitus	165	24.7
Others	140	21.0
Antihypertensive use		
Yes	444	66.6
Parathyroidectomy		
Yes	63	9.5
Hepatitis B		
Yes	92	13.8
Hepatitis C		
Yes	49	7.4
24 hr urinary volume(ml)		
mean, SD	0.61	0.64

Abbreviations: CV, cardiovascular.

Supplementary Table 2 Baseline laboratory data in study cohort

Laboratory parameters	mean	SD
Hemoglobin(g/dl)	10.3	1.4
WBC(x1000/ul)	7.1	2.5
Glucose(mg/dl)	119.5	52.1
Albumin(gm/dl)	3.77	0.39
AST(IU/L)	22.0	11.8
ALT(IU/L)	21.4	15.9
T-BIL(mg/dl)	0.37	0.15
BUN(mg/dl)	123.5	60.7
Creatinine(mg/dl)	11.0	3.0
Ca(mg/dl)	9.5	0.8
Phosphate(mg/dl)	5.2	1.3
Sodium(meq/l)	135.3	4.4
Potassium(meq/l)	4.1	0.7
Cholesterol(mg/dl)	188.8	44.4
Triglyceride(mg/dl)	159.7	113.7
Uric acid(mg/dl)	6.9	1.3
Ferritin(ng/ml)	441.9	529.5
iPTH(pg/ml)	308.6	441.1
CTR(%)	0.48	0.06
nPCR(gm/Kg/day)	1.02	0.24
Kt/V (total)	2.08	0.49
Ccr (renal)	20.9	24.7
Ccr (total)	66.8	22.5

Abbreviations: WBC, leukocyte count; AST, aspartate sminotransferase; ALT, alanine aminotransferase; T-BIL, total bilirubin; BUN, blood urea nitrogen; Ca, albumin-corrected calcium; iPTH, intact parathyroid hormone, CTR, cardiothoracic ratio; nPCR, normalized protein catabolic rate; Ccr, creatinine clearance cc/min/1.73m^2/week
